# Enhancing Patient Engagement in HTA: Using Consensus Research to Overcome PICO Scoping Challenges Under the EU HTAR

**DOI:** 10.3390/jmahp13020027

**Published:** 2025-06-02

**Authors:** Emanuele Arcà, Adele Barlassina, Adaeze Eze, Valentina Strammiello

**Affiliations:** 1 OPEN Health, Marten Meesweg 107, 3068 AV Rotterdam, The Netherlandsadaezeeze@openhealthgroup.com (A.E.); 2 European Patients’ Forum, Chaussée d’Etterbeek 180, 1040 Brussels, Belgium

**Keywords:** patient engagement, EU HTA Regulation, PICO scoping, consensus research, Delphi panels, Health Technology Assessment

## Abstract

The evolving landscape of Health Technology Assessment (HTA) in Europe, shaped by the implementation of the new EU HTA Regulation (HTAR), places an emphasis on engaging all stakeholders, including patients, in collaborative evidence generation. Yet integrating patients’ perspectives into critical processes like PICO scoping remains a challenge, with concerns around subjectivity, representativeness, and methodological robustness. This opinion paper examines the complexities of patient engagement in HTA, highlighting both the opportunities for patients to make meaningful contributions and the barriers that stand in the way. We propose a framework that employes the Delphi panel methodology to (1) foster scientific validity and increase transparency in patient contributions, (2) establish a structured and consistent patient engagement framework, and (3) and understand European patients’ perspectives while promoting collaboration among EU countries. By facilitating iterative feedback and fostering agreement among diverse groups of patients and caregivers contributing with their expertise, consensus methods like Delphi panels can help refine PICO criteria, align diverse stakeholders’ expectations, and increase the relevance of HTA outcomes. A study is now underway to evaluate the feasibility and value of using the modified Delphi panel methodology for patient engagement in PICO scoping. The authors propose that embracing patient engagement through carefully designed consensus frameworks could enhance the legitimacy and completeness of HTA processes, driving more patient-centered decision making across Europe.

## 1. Introduction

The aim of the European Union (EU) regulation on Health Technology Assessment (HTA), which entered into force on 12 January 2022 (applicable as of 12 January 2025), is to harmonize HTA processes across EU Member States, reduce the administrative burden on industry and national authorities, and increase the quality of HTA and evidence-based decision making. The HTA Regulation (HTAR) provides a permanent framework for Joint Scientific Consultations (JSCs) and Joint Clinical Assessments (JCAs) at the EU level, ensuring a harmonized, evidence-based approach to HTA for new medicines and certain high-risk medical devices [1]. The JSC allows health technology developers to seek scientific advice from HTA bodies to ensure that the evidence generated meets the requirements for a JCA. Patient input can help shape the research questions and outcomes measured to ensure the research addresses patients’ needs [2,3].

A key component of the HTAR is the population, intervention, comparator, and outcome (PICO) framework. Its aim is to ensure that comparative clinical evidence meets the needs of all EU Member States; as such, PICO forms the foundation of JCAs. The regulation mandates that each JCA be structured around consolidated PICO definitions that reflect input from all EU countries and diverse stakeholders to enable an inclusive, standardized, and transparent assessment of health technologies. To ensure that the needs of all EU Member States and other stakeholders are considered, a survey is conducted by the assigned assessors and co-assessors to gather input on PICO definitions. The collected responses are then consolidated by the Coordination Group [1], with the aim to minimize the number of distinct PICO frameworks while maintaining relevance across jurisdictions. However, there is a concern that, given variations in clinical practice among Member States, multiple PICOs will be required for most JCAs [1].

Another key aspect of the regulation is an emphasis on patients as stakeholders in the HTAR process and a recognition of the importance of incorporating their perspectives into the assessment and regulatory process at an EU-wide level. Thus, early engagement with patient organizations including patient advocacy groups (PAGs) is necessary for the appropriate representativeness of all stakeholders in the HTA process [4,5].

However, there are several barriers to patients’ active participation in JSCs and JCAs [6]. Extensive effort and a distinct research agenda, such as establishing clear processes and pathways for stakeholder engagement in PICO scoping, are required to ensure adequate involvement of key stakeholder groups, including patients, in the evolving EU HTA process [7].

This paper discusses the role of patient engagement in HTA, addressing some of the challenges and opportunities presented by the HTAR, with a primary focus on PICO scoping. Additionally, the paper offers insights into how consensus research methods, such as Delphi panels, could help address some of these challenges.

While Delphi methods have been used in healthcare research and stakeholder engagement, their application to structuring and formalizing patient input into the PICO development process within the context of the EU HTA is, to our knowledge, novel. We propose a Delphi-based framework to integrate patient perspectives systematically and transparently during the early scoping phase, an area still underdeveloped in the EU HTA practice.

Furthermore, by involving patients in structured consensus-building exercises across multiple Member States, the Delphi approach can help reduce heterogeneity in PICO definitions, while also identifying and exploring legitimate differences in preferences, values, and contextual needs. This dual function—aggregating opinions and illuminating dissent—is particularly valuable in the EU context, where cross-country variability poses a challenge to JCAs.

Overall, this represents an innovative contribution to the methodological landscape of the EU HTA by promoting both inclusivity and analytical depth in early evidence planning.

## 2. Engaging Patients in HTA

Integrating patients’ perspectives into HTA processes can improve the relevance and quality of HTA outcomes, ensure that assessments reflect patients’ needs and preferences, and enhance transparency and trust in the HTA process [8,9]. (See Table 1, The Importance of Patient Engagement in HTA.)

Broadly speaking, the key areas of patient involvement in the HTA process are as follows [10]:

- Scoping phase: Patients can identify outcomes and endpoints that are relevant from their perspective, highlight unmet needs, and pinpoint areas where healthcare interventions require improvement.
- Evidence generation: Patients can provide insights into the real-world impact and value of health technologies via patient-reported outcomes and insights gleaned from their lived experience.
- Assessment and appraisal: Patients can review draft reports, offer feedback, and participate in discussions and decision-making processes, helping to align HTA findings with patient needs and expectations.

Patient organizations and patient advocacy groups (PAGs) serve as essential facilitators to engage patients and integrate their perspectives in the HTA process. These groups represent patients in policy discussions, identify relevant patient experts, and provide training to empower meaningful participation. Their contributions are instrumental in bridging the gap between technical evaluations and patient-centered outcomes, ultimately enhancing the quality and relevance of an HTA [11,12].

In a qualitative analysis by the Patient and Citizen Involvement in HTA Interest Group, a three-domain framework approach was proposed to categorize the impact of patient involvement in HTA [13]. This includes the impact on the basis of HTA results or recommendations, impact on HTA body, and impact on patient participants. The analysis showed that the framework may be useful in identifying, evaluating, and communicating the value of patient involvement in HTA. A retrospective study analyzed how the French patient and consumer groups (PCGs) contributed to the HTA process within the HAS via a process called the systematic open e-contribution (SOEC). The results demonstrated the feasibility of the process and confirmed that PCG contributions provide relevant insights into the patient perspective for HTAs used in reimbursement decisions [14]. These studies underscore the critical role of patient engagement in HTA.

**Table 1:** The Importance of Patient Engagement in HTA.

As the recipients of health technologies, patients possess a unique perspective on HTA processes. Specifically, patients offer the following:
Unique insights into diseases and treatments, enriching the understanding of the real-world impact of diseases and interventions;
Identification of unmet needs, allowing the optimization of clinical study designs by highlighting important considerations such as patient-relevant outcomes crucial to their quality of life, selecting appropriate comparators, well-defined inclusion/exclusion criteria, and strategies to mitigate practical challenges associated with patient recruitment, retention, or treatment burden;
Alignment with patient values and preferences, enhancing the relevance and acceptability of health technologies and medicinal products [8,9,15,16].

## 3. Challenges with Patient Engagement in HTA

Several frameworks have been developed to standardize and optimize patient engagement in HTA across the globe [17], with relevant examples in Canada [18] and Brazil [19] or specific to certain disease areas like rheumatology [19]. Despite these efforts, significant challenges remain in effectively integrating patient preferences into HTA processes [20,21]. These challenges can be categorized as follows:

- Methodological and procedural barriers: These include managing preference heterogeneity, selecting appropriate methods for preference elicitation, and evaluating the impact of preference studies on decision-making outcomes [22].
- Gaps in support mechanisms: There are persistent gaps in the structural support for patient involvement in HTA. These include a lack of appropriate supporting mechanisms for patients and patient organizations, providing clarity on patient roles, and ensuring a consistent flow of information among patients, developers, and assessors [21].
- Variation in reporting standards: The comprehensiveness and methodological transparency of HTA reports can vary significantly, and inconsistent reporting standards undermine the reliability and reproducibility of patient-related assessments, ultimately limiting the meaningful integration of patient preferences into HTA decision making [23,24,25].
- Challenges in patient representativeness: The representativeness of patients in healthcare decision making remains a contentious issue for assessors and developers. Divergent conceptualizations of what constitutes legitimate representation often lead to disagreements among assessors, patients, and developers, complicating efforts to obtain equitable and meaningful patient involvement [26].
- Limited integration of patient perspectives: HTA processes remain primarily focused on quantitative evidence to assess the clinical benefits and cost effectiveness of health technologies. As a result, patient perspectives on their condition and the impact of a Health Technology Assessment are rarely considered or are perceived as subjective or biased. Yet, incorporating structured and validated patient input could enhance the relevance and completeness of HTA evaluations, provided reliable mechanisms for systematic engagement are in place [27].
- Capacity and capability: Relevant patients are often unaware of opportunities to become involved, lack the knowledge and resources to contribute effectively, and are uncertain about how to participate in the HTA process [28].

## 4. Opportunities for Patient Engagement Under the New EU HTAR

**Figure 1:**
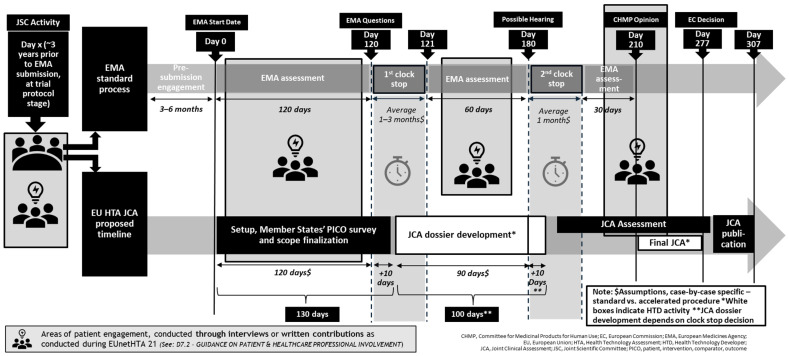
Patient engagement within the EU HTA process.

The HTAR offers an opportunity to integrate structured patient input into the HTA process, but it also introduces several challenges for patient engagement in terms of timelines and patients’ capacity. Figure 1 provides an overview of all relevant patient timepoints across the HTAR process. On top of the traditional engagements with the European Medicines Agency across the regulatory process, patient organizations and PAGs need to simultaneously engage in the EU HTA process. The HTAR mandates the systematic and timely involvement of patient experts, ensuring their active participation in key activities such as JSCs and JCAs. In particular, as detailed in Figure 1, the regulation outlines where patient involvement is mandated:

- During the preparation of the final JSC outcome document, the designated subgroup ensures that patients, clinical experts, and other experts are given an opportunity to provide input.
- During the PICO scoping process, input from patients, clinical experts, and other experts is to be taken into account by the assessors and co-assessors.
- During the clinical assessment process, the designated assessors and co-assessors ensure that patients, clinical experts, and other relevant experts are involved by giving them the opportunity to provide input on draft reports. This input must be provided within the established framework and timelines [1].

The tight JCA timelines (completion within 130 days from the Committee for Medicinal Products for Human Use—CHMP—opinion publication) leaves limited time for meaningful engagement with patients (Figure 1). Moreover, meaningful engagement with patients during the JCA process may be challenging, as patients may not be familiar with the JCA concept and process [6].

In addition to timeline and technical challenges, there are the issues of representation and conflicts of interest. Ensuring that patient experts represent the broader patient population and are not simply reflecting the views of specific organizations or individual experiences is critical for equitable input. Additionally, conflicts of interest—arising from affiliations with industry, from connections with advocacy groups, or from funding dependencies—must be transparently managed to uphold the integrity and impartiality of the HTA process [21].

The HTAR does not provide clear guidance on how patients should be involved in the JSC and JCA process. This has led to concerns that patient engagement may be superficial and that patient views may not be adequately considered. Compiling a harmonized PICO, adapting local procedures, and increasing the capacity to actively take part in the JSC and JCA are seen as primary barriers by several stakeholders, including patients [6].

### 4.1. Patient Engagement in PICO Scoping

**Figure 2:**
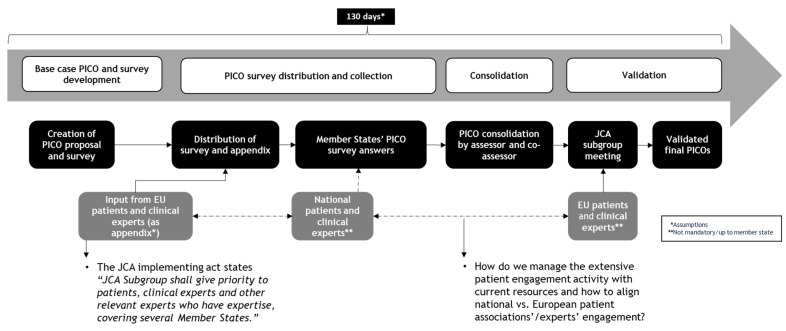
Patient engagement within the PICO scoping process.

The PICO framework—defining population, intervention, comparator, and outcomes—is central to structuring the assessment of clinical evidence, guiding the formulation of research questions, and determining the evidence synthesis strategy. Within the new HTAR, patient organizations and PAGs are allocated three months to contribute to PICO scoping, first at the European level and then within national contexts (Figure 2).

While this structured and time-constrained process enhances alignment across jurisdictions, it also introduces significant challenges for meaningful and timely patient input. Hereafter, we explore the complexities of patient engagement in PICO scoping and propose potential solutions to optimize their involvement within this tight, dynamic framework.

#### 4.1.1. PICO Scoping Methods and Patients’ Role

Both at the national and EU levels, there is no harmonization of approaches for obtaining patient input into the development of PICOs. It is yet not clear how priority should be given to subject matter experts who act in an individual capacity and who have expertise in the therapeutic area of interest from a European or international perspective [29] nor how conflicts of interest could be managed, particularly in disease areas with a limited number of patient experts.

Moreover, the absence of standardized methodologies for patient involvement across Member States and the lack of clear mechanisms for integrating patient experience studies into the JCA process are significant gaps that further complicate efforts to ensure consistency and inclusivity. Compounding these challenges are tight timelines, which hinder the timely identification and meaningful involvement of patients.

#### 4.1.2. National vs. EU-Level Discrepancies

At the intersection of national and EU-level HTA processes, additional complexities arise. There is a potential disconnect between European-level and national-level patient organizations and PAGs. Many national patient organizations lack the knowledge, human resources, and financial capacity to effectively participate in the JCA process.

Furthermore, the absence of formalized methods for patient involvement at the national level by HTA bodies increases the risk of fragmented or incoherent feedback across EU and national processes. This fragmentation, combined with variability in the personalization of PICOs, risks making patient input less comparable, consistent, and impactful [30]. Without harmonized and well-adapted PICOs, patient input might become fragmented, harder to compare, and less likely to be meaningfully used in HTA decision making.

### 4.2. Strategies to Enhance Patient Engagement Within EU HTA

To address the challenges mentioned above, several strategies should be considered. The European Patients’ Forum (EPF) and 10 other European PAGs developed 10 key recommendations [31] to promote meaningful and practical patient involvement (Table 2)).

These 10 recommendations stress the need for clear guidance to be developed to define the roles and responsibilities of patients in the JCA process. Moreover, resources must be allocated to support patient engagement, including funding for POs to actively participate in the JCA process and contribute to evidence generation and assessment. Finally, innovative approaches to patient engagement should be prioritized, including the development of scientifically robust methodological guidelines for capturing patient preferences and insights through preference studies and consensus-based research, with findings disseminated in peer-reviewed publications.

In the authors’ opinion, consensus research methods, such as Delphi panels, may help to address many of these challenges while increasing the transparency and inclusiveness of the process. Section 5 provides a more detailed discussion of the potential use of consensus research in HTA.

## 5. Consensus Research, Including Delphi Panels, for Stakeholder Engagement in HTA

Consensus research methodologies, such as Delphi panels, are a recognized and valid approach for stakeholder engagement in health policy making due to their ability to integrate diverse perspectives, foster stakeholder participation, and enhance the legitimacy and acceptability of policy decisions [32,33,34]. These methodologies foster a structured environment in which stakeholders, including patients, can collaboratively identify, discuss, and prioritize policy options [32,33,34]. Furthermore, these methods support transparent, accountable, and rigorous decision-making processes and give voice to traditionally under-represented groups, such as patients [35,36]. Engagement through a structured and transparent approach enhances the legitimacy of stakeholder involvement, as it is well supported by peer-reviewed literature and has a demonstrated track record in healthcare decision making [37,38,39].

Transparency, inclusivity, the integration of diverse perspectives, and legitimacy are also key principles highlighted by Health Technology Assessment International (HTAi) and the ISPOR Task Force on deliberative processes in HTA. The task force emphasized the following:

- Transparency and inclusivity as foundational principles guiding HTA deliberations;
- Value-based discussions that allow for open dialogue among those with differing and potentially conflicting perspectives;
- Legitimacy of both the deliberative process and its outcomes as essential goals.

We believe that consensus methodologies are particularly well-suited to advancing stakeholder engagement in HTA and aligning with the deliberative process requirements outlined by the task force.

Further supporting the suitability of consensus methods for stakeholder engagement in HTA, consensus research is increasingly being applied in health economics, particularly through expert elicitation methods. In the UK, Structured Expert Judgment using the SHELF method has been widely adopted to elicit and synthesize expert opinions, especially for parameter estimation and managing uncertainty in health economic models [40]. Similarly, in the US, the Innovation and Value Initiative has shown that engaging a broad advisory group in model development can provide valuable insights and improve the design of economic models, even if practical challenges in implementation remain [41]. Although engagement can promote mutual understanding and collaboration, it is essential to acknowledge that consensus may not always be achieved and to manage disagreements transparently and constructively [42].

## 6. Integrating Patient Voices in PICO Scoping Through Delphi Panels: A Framework Proposal

EPF and OPEN Health (OH) are jointly conducting a study to evaluate the feasibility, reliability, and efficiency of a framework employing a modified Delphi panel methodology for incorporating patient perspectives into PICO scoping.

With this study, EPF and OH aim to achieve the following:

- Assess the appropriateness of consensus methods for stakeholder engagement in PICO scoping;
- Identify best practices for collaboration among European and national patient organizations and PAGs;
- Evaluate the variability in patient input into PICO scoping across Europe.

### 6.1. Selection of Relevant PAG and Disease Area for Testing

EPF and OH first shortlisted three PAGs within the EPF’s network for collaboration. The choice of the PAG was guided by two considerations: (1) disease area, with priority given to rare diseases where data on patient outcomes are often scarce, and (2) therapy type, with a focus on innovative therapies such as gene therapies due to their profound, life-altering potential, making the inclusion of patient perspectives in PICO scoping particularly crucial.

The European Alliance of Neuromuscular Disorders Association (EAMDA) agreed to collaborate and contributed to the decision to run the PICO scoping simulation for onasemnogene abeparvovec (Zolgensma) for children up to the age of 9 years old with spinal muscular atrophy (SMA). Onasemnogene abeparvovec is a one-time gene therapy approved for treating patients up to 2 years of age who have SMA caused by the *SMN1* gene mutation.

### 6.2. Implementation of the Modified Delphi Method

**Figure 3:**
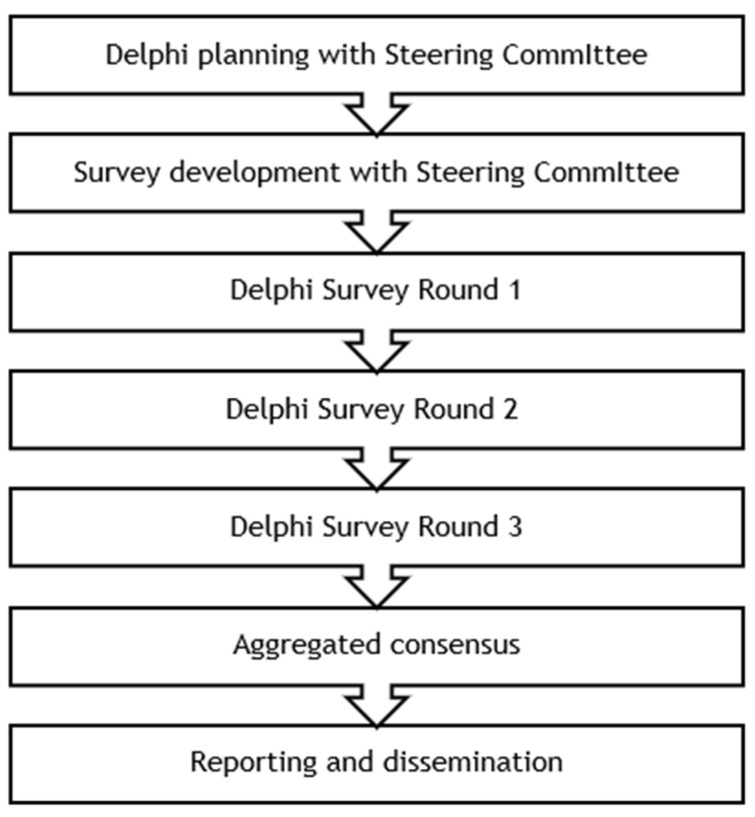
Modified Delphi method to be employed.

This study employs a three-round modified Delphi method (Figure 3). A steering committee consisting of two clinicians selected based on their expertise in SMA, one patient representative of EAMDA, and an HTA policy expert from the EPF informed the selection criteria for the Delphi panelists and the development of the consensus survey. The EPF and EAMDA collaborated in recruiting panelists from their networks of patients and patient representatives. Twelve individuals who were either patients with SMA or caregivers of patients with SMA from 12 EU countries consented to participate as panelists in the Delphi study. These individuals were selected based on whether they were an active member of an SMA patient organization and whether they had experience representing patients’ perspectives, for instance through participation in research projects, policy discussions, or conference presentations. Collaborating with the EAMDA from study inception allowed the selection of patient representatives with highly relevant profiles across a broad geography.

By this study’s end, panelists will have completed the consensus survey three consecutive times. It includes structured opinion statements presented to panelists with a 5-point Likert response scale (“Strongly agree,” “Agree,” “Neither agree nor disagree,” “Disagree,” and “Strongly disagree”) with the addition of “I don’t know” and open-ended questions.

### 6.3. Expected Impact

**Figure 4:**
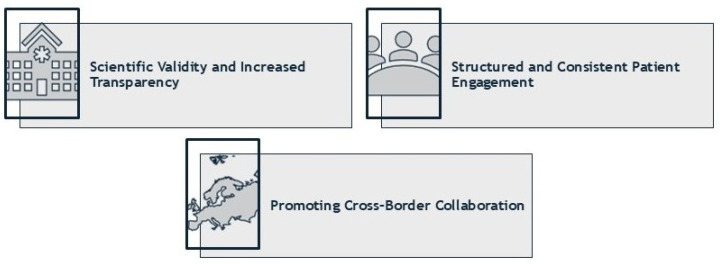
Intended benefits of including patients in PICO scoping through a Delphi consensus exercise.

We anticipate that implementing a patient engagement framework based on a modified Delphi panel methodology for PICO scoping will provide several key benefits. As outlined in Section 5, Delphi panels are a scientifically validated approach for fostering stakeholder collaboration in policy development, aligning with the recommendations of the HTAi and ISPOR Task Forces on deliberative processes in HTA. We expect a patient engagement framework based on the modified Delphi panel methodology to (1) foster scientific validity and increases transparency, (2) establish a structured and consistent patient engagement framework, and (3) promote collaboration across EU countries, ultimately strengthening the European HTA landscape and supporting equitable access to innovation (Figure 4). The expected benefits and anticipated challenges of patient engagement in PICO scoping are presented in detail below.

#### 6.3.1. Fostering Scientific Validity and Increasing Transparency

A key advantage of the modified Delphi methodology is its ability to integrate diverse stakeholder input in a systematic, transparent, and reproducible manner. By leveraging an iterative process with controlled feedback, it allows for a structured convergence of experts’ and patients’ opinions, minimizing bias and promoting robust scientific validity. The anonymity of Delphi panels further reduces the influence of dominant voices, ensuring that all perspectives—regardless of hierarchy, institutional affiliation, or geographical representation—are equally considered [43].

In addition to the statistical aggregation of responses, the modified Delphi approach facilitates the qualitative analysis of stakeholder comments, providing deeper insights into areas of agreement and contention [44]. This structured methodology ensures that patient-relevant considerations in PICO scoping are not merely anecdotal but are assessed with the same level of rigor as other expert inputs. Moreover, its ability to produce consensus-based recommendations supports the creation of a transparent and scientifically sound foundation for decision making, ultimately contributing to peer-reviewed publications that strengthen the HTA process.

#### 6.3.2. Establishing a Structured and Consistent Patient Engagement Framework

Despite the growing recognition of the importance of patient engagement in HTA, current approaches remain inconsistent across jurisdictions, leading to the fragmented and ad hoc inclusion of patient perspectives. A modified Delphi panel provides a formalized and structured mechanism for systematically collecting stakeholder input early in the HTA process, covering a broad range of EU countries. By requiring structured participation from patients’ representatives and other relevant stakeholders, this methodology ensures that patient needs and societal considerations are embedded in PICO requirements early on.

Furthermore, this structured engagement might enhance the predictability and appropriateness of HTA outcomes. By enlarging the pool of systematically gathered information, the Delphi process helps anticipate areas of uncertainty and allows for proactive mitigation strategies. This is particularly relevant in the context of rare diseases and innovative therapies, where patient perspectives can provide critical insights into unmet needs and treatment priorities that may not be fully captured through traditional clinical evidence alone.

#### 6.3.3. Understanding European Patients’ Input and Promoting Cross-Border Collaboration

HTA systems across Europe operate within distinct national frameworks, often resulting in variations in evidence requirements, access pathways, and stakeholder engagement approaches. The modified Delphi method proposed here not only facilitates a structured consensus-building process but also provides a nuanced understanding of areas of agreement, convergence, dissent, and divergence among European patients. By systematically capturing differing perspectives, this approach ensures that variations in patient needs and priorities across countries are acknowledged and addressed, ultimately contributing to more inclusive and representative decision making in HTA.

The implementation of a modified Delphi panel at the European level could also serve as a unifying mechanism to foster cross-border collaboration and consistency in patient engagement across national HTA bodies. By enabling diverse European stakeholders to engage in a structured and iterative consensus-building process, the Delphi methodology contributes to a more harmonized approach to HTA. This is particularly relevant in the evolving European access landscape, where JCAs under the new EU HTAR will require increased coordination among Member States. A standardized Delphi framework could help build trust among national HTA agencies, facilitate the development of common methodologies, and ultimately support the establishment of a more predictable and equitable system for access across Europe.

#### 6.3.4. Anticipated Challenges of the Delphi Framework for Patient Engagement in PICO Coping

In the early stages of this study, several barriers to implementing a Delphi framework for patient engagement in PICO scoping were identified. One is defining what constitutes patient expertise, thereby to ensure legitimate representativeness. To address this, collaboration with European PAGs is proposed so that they can leverage their national networks to identify and recruit qualified patient experts with appropriate representational legitimacy. Additionally, establishing a Delphi steering committee to oversee the development and implementation of this study can provide critical guidance in defining patient expertise relevant to the specific PICO scoping exercise and help ensure that high scientific standards are maintained throughout the process. Another barrier is the diversity across European healthcare systems, which affects how patients from different countries may perceive or prioritize elements within the PICO framework. To ensure the Delphi survey is relevant and applicable across diverse settings, the approach includes close collaboration with national PAGs to validate and adapt the survey content accordingly. Finally, recognizing the limited resources often available to PAGs, this study emphasizes the importance of demonstrating the validity and impact of the Delphi approach. By achieving this, it seeks to highlight the added value of PAGs in identifying appropriate patients and supporting meaningful engagement throughout the Delphi process.

## 7. Conclusions

The implementation of the new EU HTAR presents a unique opportunity to strengthen patient engagement in HTA processes. However, without a structured and consistent approach, there is a risk that patient input will remain fragmented, ad hoc, or merely symbolic. To ensure that patient perspectives meaningfully inform decision making, it is critical to adopt methodologies that facilitate structured, transparent, and iterative stakeholder engagement.

This paper has explored the challenges and opportunities of patient engagement in HTA, with a particular focus on PICO scoping under the new HTAR. Through the case study of a modified Delphi panel, we have argued how consensus research methods could provide a systematic framework for integrating diverse stakeholder perspectives, including patients and clinicians. The Delphi approach could enhance scientific rigor through controlled feedback and consensus building while also fostering cross-border collaboration by aligning stakeholder input across European HTA bodies. Importantly, applying the Delphi method to PICO scoping with patient involvement represents a novel and underexplored approach. It offers a structured means to capture patient-relevant outcomes while promoting transparency and inclusiveness in decision making. Moreover, it may contribute to reducing heterogeneity in PICO definitions across Member States by surfacing shared priorities and clarifying divergent views. By aggregating opinions while also explicitly documenting areas of dissent, a patient-informed Delphi process can help HTA bodies and developers navigate the complexity of multinational evidence requirements under the EU HTAR. This approach could add methodological depth to stakeholder engagement and also support the practical implementation of a more coherent and patient-centered HTA process across Europe.

As Europe moves toward greater HTA harmonization, the success of the new regulatory framework will depend on the mechanisms put in place to facilitate inclusive and transparent stakeholder engagement. This is a key starting point for an inclusive civil society dialogue—an open exchange between policymakers, stakeholders, and the public to shape policies and decisions—as suggested by the European Commission’s Pharmaceutical Strategy [4,45]. Moreover, the recent Report on the Future of Europe by Mario Draghi highlights the need for stronger coordination in pharmaceutical policy, emphasizing HTA as a key mechanism to improve access, affordability, and innovation across European healthcare systems [46]. In this context, ensuring that HTA processes integrate diverse stakeholder perspectives—particularly those of patients—is critical to aligning regulatory frameworks with real-world healthcare needs and fostering a more resilient and equitable European health system.

## Figures and Tables

**Table 2:** Ten Recommendations for Enhancing Patient Engagement in HTA.

*1. Establish a predictable framework for patient involvement in JCAs;*
*2. Include input from patients, carers, and patient organizations;*
*3. Include patient experience data in JCAs;*
*4. Streamline patient involvement throughout the process;*
*5. Provide plain language summaries of technologies;*
*6. Broaden the pool of patients and specify selection criteria;*
*7. Provide support to patients;*
*8. Make JCA and summary reports available in all EU languages;*
*9. Provide feedback to patients;*
*10. Adopt a constructive approach to confidentiality and conflicts of interest* [31].

## Data Availability

No new data were created or analyzed in this study. Data sharing is not applicable to this article.

## Abbreviations

The following abbreviations are used in this manuscript:

EAMDA	European Alliance of Neuromuscular Disorders Association
EPF	European Patients’ Forum
EU	European Union
HTA	Health Technology Assessment
HTAi	Health Technology Assessment International
HTAR	Regulation on Health Technology Assessment
JCA	Joint Clinical Assessment
JSC	Joint Scientific Consultation
OH	OPEN Health
PAG	Patient advocacy group
PCG	Patient and consumer group
PICO	Population, intervention, comparator, outcome
SMA	Spinal muscular atrophy
